# Chlorido(4,4′,4′′-tri-*tert*-butyl-2,2′:6′,2′′-terpyridine)­platinum(II) chloride toluene monosolvate

**DOI:** 10.1107/S1600536810048750

**Published:** 2010-11-30

**Authors:** Rami J. Batrice, Vladimir N. Nesterov, Bradley W. Smucker

**Affiliations:** a Department of Chemistry, Austin College, 900 North Grand, Sherman, TX 75090-4400, USA; b Department of Chemistry, University of North Texas, 1155 Union Circle, #305070, Denton, TX 76203-5070, USA

## Abstract

In the title compound, [PtCl(C_27_H_35_N_3_)]Cl·C_7_H_8_, the Pt^II^ atom is coordinated in a pseudo-square-planar fashion by the N atoms of a 4,4′,4′′-tri-*tert*-butyl-2,2′:6′,2′′-terpyridine (tbtrpy) ligand and a Cl atom. The Pt—N distance of the N atom on the central pyridine is 1.941 (4) Å, while the peripheral N atoms have Pt—N distances of 2.015 (4) and 2.013 (4) Å. The Pt—Cl bond distance is 2.3070 (10) Å. The cations pack as dimers in a head-to-tail orientation with an inter­molecular Pt⋯Pt distance of 3.2774 (3) Å and Pt⋯N distances of 3.599 (4), 3.791 (4) and 4.115 (4) Å. The solvent mol­ecule is disordered and occupies two positions with a ratio of 0.553 (6):0.447 (6).

## Related literature

For crystal structures of the title cation, [(tbtrpy)PtCl]^+^, see: Batrice *et al.* (2010) ▶; Lai *et al.* (1999 ▶). For head-to-tail packing of related terpyridine complexes with close Pt⋯Pt distances, see: Bailey *et al.* (1995 ▶); Sengul (2004 ▶). For the synthesis of [(tbtrpy)PtCl]Cl, see: Howe-Grant & Lippard (1980 ▶).
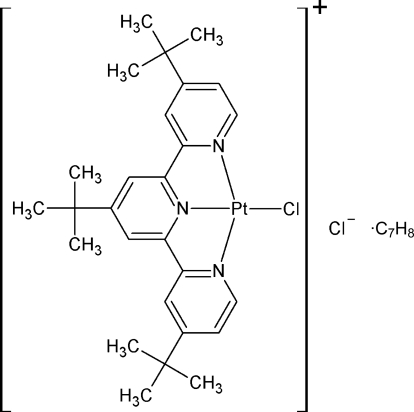


## Experimental

### 

#### Crystal data

[PtCl(C_27_H_35_N_3_)]Cl·C_7_H_8_
*M* *_r_* = 759.70
Monoclinic, 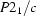

*a* = 9.4418 (3) Å
*b* = 20.0002 (7) Å
*c* = 17.2321 (6) Å
β = 91.948 (1)°
*V* = 3252.19 (19) Å^3^
*Z* = 4
Mo *K*α radiation
μ = 4.51 mm^−1^
*T* = 100 K
0.26 × 0.21 × 0.09 mm

#### Data collection

Bruker SMART APEXII CCD diffractometer
Absorption correction: multi-scan (*SADABS*; Bruker, 2007 ▶) *T*_min_ = 0.387, *T*_max_ = 0.677
37919 measured reflections
6647 independent reflections
6232 reflections with *I* > 2σ(*I*)
*R*_int_ = 0.030

#### Refinement

*R*[*F* ^2^ > 2σ(*F* ^2^)] = 0.032
*wR*(*F* ^2^) = 0.108
*S* = 1.05
6647 reflections
358 parameters
14 restraints
H-atom parameters constrained
Δρ_max_ = 1.47 e Å^−3^
Δρ_min_ = −2.78 e Å^−3^

### 

Data collection: *APEX2* (Bruker, 2007 ▶); cell refinement: *SAINT* (Bruker, 2007 ▶); data reduction: *SAINT*; program(s) used to solve structure: *SHELXS97* (Sheldrick, 2008 ▶); program(s) used to refine structure: *SHELXL97* (Sheldrick, 2008 ▶); molecular graphics: *SHELXTL* (Sheldrick, 2008 ▶) and *Mercury* (Macrae *et al.*, 2008 ▶); software used to prepare material for publication: *SHELXTL*.

## Supplementary Material

Crystal structure: contains datablocks global, I. DOI: 10.1107/S1600536810048750/bv2164sup1.cif

Structure factors: contains datablocks I. DOI: 10.1107/S1600536810048750/bv2164Isup2.hkl

Additional supplementary materials:  crystallographic information; 3D view; checkCIF report

## Figures and Tables

**Fig. 1.**
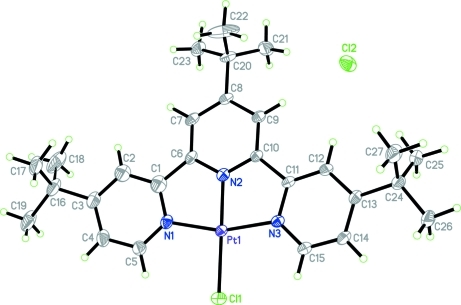
View of title complex (50% probability displacement ellipsoids) without the toluene solvate.

**Fig. 2.**
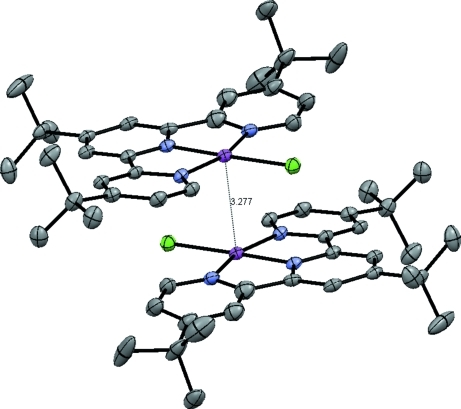
Mercury (Macrae,et al., 2008) rendition of head-to-tail packing with Pt—Pt' distance in 3.2774 (3) Å

## supplementary crystallographic information

### Comment

The bond distances and angles around the platinum atom in the title structure are all similar to the structures of the perchlorate (Lai, *et al.*, 1999) and tetrafluoroborate salts (Batrice *et al.* 2010) of the [(tbtrpy)PtCl]^+^ complex. The cations in these structures all pack in head-to-tail dimers. Interestingly, the interplanar (Pt, Cl and N atoms) distance between the two cations seems to be related to the size of the anion with the Cl^-^, BF_4_^-^, and ClO_4_^-^ being 3.283, 3.390, and 3.536 Å, respectively. In addition to a smaller counterion, the structure of the title complex contains a toluene molecule. This suggests that the solvent molecule may also influence the ability of these types of complexes to interact significantly with each other (Bailey, *et al.*, 1995).

The short Pt(1)—Pt(1') distance, 3.2774 (3) Å, of the title complex is similar to the intermolecular Pt—Pt distance in the structures of [(trpy)PtCl]Cl, 3.397 Å, (Sengul, 2004) and [(trpy)PtCl]ClO_4_, 3.269 Å, (Bailey *et al.*, 1995). This indicates that the bulky *tert*-Butyl groups of the tbtrpy ligand do not appear to restrict the ability of this complex to form suitable M—M and/or π-π interactions between the two molecules of the dimer.

The solvent molecule is disordered and occupies two positions with a ratio of 0.553 (6):0.447 (6).

### Experimental

[(tbtrpy)PtCl]Cl was synthesized according to modifications on a published procedure (Howe-Grant *et al.*, 1980). This [(tbtrpy)PtCl]^+^ complex was reacted with various aromatic thiol ligands (SAr). Crystals of the title compound were grown from the slow evaporation of an acetonitrile/toluene solution containing [(tbtrpy)Pt(SAr)]Cl and [(tbtrpy)PtCl]Cl.

### Refinement

H atoms attached to C atoms were placed in idealized positions (C—H = 0.95–0.98 Å) and allowed to ride on their parent atoms. All H atoms were constrained so that *U*_iso_(H) were equal to 1.2*U*_eq_ or 1.5*U*_eq_ of their respective parent atoms. The solvent molecule is disordered and occupies two positions with a ratio of 0.553 (6): 0.447 (6). Aromatic C atoms were fitted to a regular hexagon with default distances 1.390 Å (AFIX 66) and refined anisotropically. Both CH_3_ groups were refined anisotropically with fixed C—C distances as 1.51 Å. The largest peak in the final Fourier difference map (1.51 e Å^-3^) was located 1.40 Å from the several disordered C atoms of the solvent.

### Crystal data

**Table:**

[PtCl(C_27_H_35_N_3_)]Cl·C_7_H_8_	*F*(000) = 1520
*M**_r_* = 759.70	*D*_x_ = 1.552 Mg m^−^^3^
Monoclinic, *P*2_1_/*c*	Mo *K*α radiation, λ = 0.71073 Å
Hall symbol: -P 2ybc	Cell parameters from 9878 reflections
*a* = 9.4418 (3) Å	θ = 2.4–27.2°
*b* = 20.0002 (7) Å	µ = 4.51 mm^−^^1^
*c* = 17.2321 (6) Å	*T* = 100 K
β = 91.948 (1)°	Plate, yellow
*V* = 3252.19 (19) Å^3^	0.26 × 0.21 × 0.09 mm
*Z* = 4	

### Data collection

**Table:**

Bruker SMART APEXII CCD diffractometer	6647 independent reflections
Radiation source: fine-focus sealed tube	6232 reflections with *I* > 2σ(*I*)
graphite	*R*_int_ = 0.030
ω scans	θ_max_ = 26.4°, θ_min_ = 1.6°
Absorption correction: multi-scan (*SADABS*; Bruker, 2007)	*h* = −11→11
*T*_min_ = 0.387, *T*_max_ = 0.677	*k* = −24→24
37919 measured reflections	*l* = −21→21

### Refinement

**Table:**

Refinement on *F*^2^	Primary atom site location: structure-invariant direct methods
Least-squares matrix: full	Secondary atom site location: difference Fourier map
*R*[*F*^2^ > 2σ(*F*^2^)] = 0.032	Hydrogen site location: inferred from neighbouring sites
*wR*(*F*^2^) = 0.108	H-atom parameters constrained
*S* = 1.05	*w* = 1/[σ^2^(*F*_o_^2^) + (0.080*P*)^2^ + 5.*P*] where *P* = (*F*_o_^2^ + 2*F*_c_^2^)/3
6647 reflections	(Δ/σ)_max_ = 0.008
358 parameters	Δρ_max_ = 1.47 e Å^−^^3^
14 restraints	Δρ_min_ = −2.78 e Å^−^^3^

### Special details

**Table:**

Geometry. All e.s.d.'s (except the e.s.d. in the dihedral angle between two l.s. planes) are estimated using the full covariance matrix. The cell e.s.d.'s are taken into account individually in the estimation of e.s.d.'s in distances, angles and torsion angles; correlations between e.s.d.'s in cell parameters are only used when they are defined by crystal symmetry. An approximate (isotropic) treatment of cell e.s.d.'s is used for estimating e.s.d.'s involving l.s. planes.
Refinement. Refinement of *F*^2^ against ALL reflections. The weighted *R*-factor *wR* and goodness of fit *S* are based on *F*^2^, conventional *R*-factors *R* are based on *F*, with *F* set to zero for negative *F*^2^. The threshold expression of *F*^2^ > σ(*F*^2^) is used only for calculating *R*-factors(gt) *etc*. and is not relevant to the choice of reflections for refinement. *R*-factors based on *F*^2^ are statistically about twice as large as those based on *F*, and *R*- factors based on ALL data will be even larger.

### Fractional atomic coordinates and isotropic or equivalent isotropic displacement parameters (Å^2^)

**Table:**

	*x*	*y*	*z*	*U*_iso_*/*U*_eq_	Occ. (<1)
Pt1	0.588936 (16)	0.429628 (7)	0.502253 (9)	0.01709 (9)	
Cl1	0.80289 (11)	0.48538 (5)	0.50311 (6)	0.0234 (2)	
N1	0.5430 (5)	0.43245 (16)	0.3872 (2)	0.0212 (8)	
N2	0.4112 (3)	0.38083 (19)	0.50120 (19)	0.0192 (8)	
N3	0.5802 (4)	0.40887 (18)	0.6163 (2)	0.0176 (7)	
C1	0.4201 (6)	0.3997 (3)	0.3663 (3)	0.0343 (7)	
C2	0.3788 (6)	0.3916 (3)	0.2892 (3)	0.0343 (7)	
H2A	0.2930	0.3690	0.2759	0.041*	
C3	0.4636 (7)	0.4170 (2)	0.2303 (3)	0.0317 (11)	
C4	0.5847 (6)	0.4531 (3)	0.2542 (3)	0.0346 (12)	
H4A	0.6420	0.4734	0.2166	0.041*	
C5	0.6209 (5)	0.4591 (2)	0.3320 (3)	0.0276 (10)	
H5A	0.7043	0.4831	0.3468	0.033*	
C6	0.3424 (5)	0.3720 (2)	0.4324 (2)	0.0189 (8)	
C7	0.2114 (5)	0.3402 (2)	0.4302 (3)	0.0210 (9)	
H7A	0.1619	0.3328	0.3820	0.025*	
C8	0.1530 (4)	0.3190 (2)	0.4998 (2)	0.0190 (8)	
C9	0.2314 (4)	0.3270 (2)	0.5698 (3)	0.0197 (8)	
H9A	0.1957	0.3110	0.6171	0.024*	
C10	0.3625 (4)	0.3590 (2)	0.5688 (2)	0.0171 (8)	
C11	0.4619 (4)	0.3728 (2)	0.6359 (2)	0.0177 (8)	
C12	0.4448 (4)	0.3510 (2)	0.7101 (2)	0.0194 (8)	
H12A	0.3620	0.3267	0.7222	0.023*	
C13	0.5482 (5)	0.3639 (2)	0.7686 (2)	0.0204 (8)	
C14	0.6643 (5)	0.4025 (2)	0.7476 (3)	0.0232 (9)	
H14A	0.7348	0.4144	0.7858	0.028*	
C15	0.6773 (5)	0.4234 (2)	0.6722 (3)	0.0211 (9)	
H15A	0.7579	0.4489	0.6592	0.025*	
C16	0.4265 (8)	0.4015 (3)	0.1449 (3)	0.0423 (15)	
C17	0.4511 (8)	0.3261 (3)	0.1326 (3)	0.0464 (15)	
H17A	0.3892	0.3006	0.1661	0.070*	
H17B	0.5503	0.3153	0.1457	0.070*	
H17C	0.4296	0.3147	0.0782	0.070*	
C18	0.2729 (9)	0.4188 (3)	0.1272 (4)	0.058 (2)	
H18A	0.2554	0.4653	0.1421	0.087*	
H18B	0.2119	0.3891	0.1565	0.087*	
H18C	0.2520	0.4133	0.0715	0.087*	
C19	0.5205 (11)	0.4413 (4)	0.0910 (3)	0.072 (3)	
H19A	0.5057	0.4892	0.0992	0.109*	
H19B	0.4959	0.4299	0.0369	0.109*	
H19C	0.6201	0.4302	0.1024	0.109*	
C20	0.0069 (5)	0.2843 (2)	0.4993 (3)	0.0242 (9)	
C21	−0.0735 (5)	0.3042 (3)	0.5714 (3)	0.0385 (13)	
H21A	−0.1653	0.2814	0.5708	0.058*	
H21B	−0.0885	0.3527	0.5713	0.058*	
H21C	−0.0181	0.2914	0.6181	0.058*	
C22	0.0365 (7)	0.2091 (3)	0.5021 (5)	0.061 (2)	
H22A	−0.0532	0.1848	0.5053	0.092*	
H22B	0.0972	0.1988	0.5478	0.092*	
H22C	0.0843	0.1956	0.4550	0.092*	
C23	−0.0820 (5)	0.3035 (3)	0.4267 (3)	0.0364 (12)	
H23A	−0.1783	0.2859	0.4310	0.055*	
H23B	−0.0389	0.2846	0.3807	0.055*	
H23C	−0.0857	0.3523	0.4222	0.055*	
C24	0.5345 (5)	0.3346 (2)	0.8503 (3)	0.0256 (9)	
C25	0.3910 (5)	0.3546 (3)	0.8826 (3)	0.0318 (11)	
H25A	0.3847	0.3380	0.9359	0.048*	
H25B	0.3143	0.3352	0.8502	0.048*	
H25C	0.3824	0.4035	0.8825	0.048*	
C26	0.6550 (5)	0.3573 (3)	0.9056 (3)	0.0318 (11)	
H26A	0.6420	0.3378	0.9571	0.048*	
H26B	0.6544	0.4061	0.9095	0.048*	
H26C	0.7458	0.3424	0.8858	0.048*	
C27	0.5403 (6)	0.2585 (3)	0.8428 (3)	0.0352 (12)	
H27A	0.5393	0.2382	0.8946	0.053*	
H27B	0.6274	0.2456	0.8173	0.053*	
H27C	0.4580	0.2429	0.8117	0.053*	
Cl2	0.14340 (13)	0.24444 (6)	0.74948 (7)	0.0315 (3)	
C1A	0.9381 (7)	0.0032 (4)	0.6429 (3)	0.067 (4)	0.553 (6)
C2A	0.9481 (12)	0.0561 (3)	0.6952 (5)	0.081 (5)	0.553 (6)
H2AA	0.9471	0.1008	0.6768	0.097*	0.553 (6)
C3A	0.9594 (12)	0.0434 (4)	0.7745 (4)	0.068 (4)	0.553 (6)
H3AA	0.9662	0.0795	0.8103	0.081*	0.553 (6)
C4A	0.9608 (9)	−0.0221 (5)	0.8015 (3)	0.050 (4)	0.553 (6)
H4AA	0.9686	−0.0307	0.8557	0.060*	0.553 (6)
C5A	0.9509 (11)	−0.0749 (3)	0.7492 (5)	0.049 (3)	0.553 (6)
H5AA	0.9518	−0.1197	0.7676	0.059*	0.553 (6)
C6A	0.9395 (10)	−0.0623 (3)	0.6699 (4)	0.059 (3)	0.553 (6)
H6AA	0.9327	−0.0984	0.6342	0.070*	0.553 (6)
C7A	0.9269 (10)	0.0104 (4)	0.5583 (4)	0.0343 (7)	0.553 (6)
H7AA	0.8482	−0.0170	0.5378	0.051*	0.553 (6)
H7AB	1.0154	−0.0045	0.5357	0.051*	0.553 (6)
H7AC	0.9097	0.0574	0.5449	0.051*	0.553 (6)
C1B	0.9217 (9)	0.0531 (4)	0.6741 (5)	0.067 (4)	0.447 (6)
C2B	0.9072 (14)	−0.0135 (5)	0.6522 (5)	0.081 (5)	0.447 (6)
H2BA	0.8806	−0.0245	0.6001	0.097*	0.447 (6)
C3B	0.9319 (16)	−0.0640 (4)	0.7064 (7)	0.068 (4)	0.447 (6)
H3BA	0.9220	−0.1095	0.6914	0.081*	0.447 (6)
C4B	0.9709 (13)	−0.0479 (5)	0.7826 (6)	0.050 (4)	0.447 (6)
H4BA	0.9877	−0.0824	0.8197	0.060*	0.447 (6)
C5B	0.9853 (13)	0.0187 (5)	0.8046 (4)	0.049 (3)	0.447 (6)
H5BA	1.0120	0.0297	0.8567	0.059*	0.447 (6)
C6B	0.9607 (12)	0.0692 (4)	0.7504 (5)	0.059 (3)	0.447 (6)
H6BA	0.9706	0.1147	0.7654	0.070*	0.447 (6)
C7B	0.8956 (12)	0.1064 (5)	0.6175 (6)	0.0343 (7)	0.447 (6)
H7BA	0.9856	0.1203	0.5959	0.051*	0.447 (6)
H7BB	0.8520	0.1446	0.6431	0.051*	0.447 (6)
H7BC	0.8318	0.0901	0.5757	0.051*	0.447 (6)

### Atomic displacement parameters (Å^2^)

**Table:**

	*U*^11^	*U*^22^	*U*^33^	*U*^12^	*U*^13^	*U*^23^
Pt1	0.01831 (12)	0.01521 (12)	0.01768 (12)	−0.00046 (5)	−0.00049 (7)	−0.00152 (5)
Cl1	0.0202 (5)	0.0217 (5)	0.0284 (5)	−0.0019 (4)	0.0007 (4)	0.0000 (4)
N1	0.030 (2)	0.0149 (18)	0.0188 (19)	0.0005 (13)	0.0009 (16)	−0.0026 (13)
N2	0.023 (2)	0.0140 (18)	0.0207 (19)	0.0014 (13)	−0.0033 (15)	0.0005 (12)
N3	0.0180 (17)	0.0145 (16)	0.0199 (17)	−0.0012 (14)	−0.0036 (14)	0.0012 (14)
C1	0.0421 (19)	0.0285 (16)	0.0320 (16)	0.0018 (14)	−0.0020 (14)	0.0033 (13)
C2	0.0421 (19)	0.0285 (16)	0.0320 (16)	0.0018 (14)	−0.0020 (14)	0.0033 (13)
C3	0.052 (3)	0.020 (2)	0.022 (2)	−0.005 (2)	−0.006 (2)	0.0014 (19)
C4	0.058 (3)	0.024 (2)	0.022 (2)	−0.006 (2)	0.006 (2)	−0.0009 (19)
C5	0.035 (3)	0.023 (2)	0.025 (2)	−0.0054 (19)	0.0051 (19)	−0.0019 (18)
C6	0.022 (2)	0.0164 (19)	0.0178 (19)	0.0026 (16)	−0.0045 (16)	−0.0031 (15)
C7	0.024 (2)	0.0129 (19)	0.026 (2)	0.0012 (16)	−0.0086 (17)	−0.0027 (16)
C8	0.017 (2)	0.0133 (19)	0.026 (2)	0.0001 (16)	−0.0061 (16)	−0.0029 (16)
C9	0.018 (2)	0.016 (2)	0.025 (2)	0.0008 (16)	−0.0044 (16)	−0.0028 (16)
C10	0.018 (2)	0.0146 (19)	0.0185 (19)	0.0003 (15)	−0.0031 (15)	−0.0009 (15)
C11	0.0162 (19)	0.0140 (19)	0.023 (2)	−0.0018 (15)	−0.0022 (16)	−0.0024 (15)
C12	0.020 (2)	0.0142 (19)	0.024 (2)	−0.0014 (16)	−0.0033 (16)	−0.0012 (16)
C13	0.022 (2)	0.020 (2)	0.019 (2)	0.0005 (16)	−0.0052 (16)	−0.0010 (16)
C14	0.025 (2)	0.021 (2)	0.023 (2)	−0.0040 (18)	−0.0066 (17)	−0.0028 (17)
C15	0.021 (2)	0.019 (2)	0.024 (2)	−0.0023 (16)	−0.0040 (18)	−0.0015 (16)
C16	0.085 (5)	0.024 (3)	0.018 (2)	−0.020 (3)	−0.005 (3)	0.000 (2)
C17	0.085 (5)	0.030 (3)	0.025 (3)	−0.018 (3)	0.009 (3)	−0.006 (2)
C18	0.098 (6)	0.037 (3)	0.037 (3)	−0.009 (3)	−0.031 (4)	0.002 (3)
C19	0.150 (8)	0.052 (4)	0.016 (3)	−0.061 (5)	0.006 (4)	0.000 (3)
C20	0.017 (2)	0.019 (2)	0.036 (2)	−0.0056 (17)	−0.0070 (18)	−0.0045 (18)
C21	0.023 (2)	0.060 (4)	0.032 (3)	−0.010 (2)	−0.003 (2)	0.005 (2)
C22	0.038 (3)	0.020 (3)	0.124 (7)	−0.009 (2)	−0.016 (4)	−0.002 (3)
C23	0.021 (2)	0.057 (4)	0.031 (3)	−0.009 (2)	−0.005 (2)	−0.010 (2)
C24	0.031 (2)	0.024 (2)	0.021 (2)	−0.0055 (18)	−0.0064 (18)	0.0030 (18)
C25	0.034 (3)	0.042 (3)	0.019 (2)	−0.008 (2)	−0.0038 (19)	0.000 (2)
C26	0.036 (3)	0.038 (3)	0.021 (2)	−0.006 (2)	−0.0103 (19)	0.006 (2)
C27	0.051 (3)	0.026 (3)	0.027 (2)	−0.004 (2)	−0.015 (2)	0.010 (2)
Cl2	0.0288 (6)	0.0344 (6)	0.0309 (6)	−0.0016 (5)	−0.0036 (4)	0.0147 (5)
C1A	0.078 (8)	0.071 (8)	0.051 (6)	−0.039 (8)	−0.012 (6)	0.013 (5)
C2A	0.108 (12)	0.087 (10)	0.047 (6)	−0.017 (9)	−0.014 (7)	−0.031 (6)
C3A	0.049 (7)	0.065 (7)	0.090 (11)	−0.009 (5)	0.001 (6)	−0.052 (8)
C4A	0.022 (4)	0.087 (12)	0.043 (6)	0.032 (5)	0.017 (4)	0.013 (7)
C5A	0.042 (6)	0.052 (6)	0.055 (6)	0.007 (4)	0.007 (5)	0.009 (5)
C6A	0.042 (6)	0.049 (6)	0.086 (9)	−0.014 (4)	0.018 (6)	−0.024 (5)
C7A	0.0421 (19)	0.0285 (16)	0.0320 (16)	0.0018 (14)	−0.0020 (14)	0.0033 (13)
C1B	0.078 (8)	0.071 (8)	0.051 (6)	−0.039 (8)	−0.012 (6)	0.013 (5)
C2B	0.108 (12)	0.087 (10)	0.047 (6)	−0.017 (9)	−0.014 (7)	−0.031 (6)
C3B	0.049 (7)	0.065 (7)	0.090 (11)	−0.009 (5)	0.001 (6)	−0.052 (8)
C4B	0.022 (4)	0.087 (12)	0.043 (6)	0.032 (5)	0.017 (4)	0.013 (7)
C5B	0.042 (6)	0.052 (6)	0.055 (6)	0.007 (4)	0.007 (5)	0.009 (5)
C6B	0.042 (6)	0.049 (6)	0.086 (9)	−0.014 (4)	0.018 (6)	−0.024 (5)
C7B	0.0421 (19)	0.0285 (16)	0.0320 (16)	0.0018 (14)	−0.0020 (14)	0.0033 (13)

### Geometric parameters (Å, °)

**Table:**

Pt1—N2	1.941 (4)	C21—H21A	0.9800
Pt1—N3	2.013 (4)	C21—H21B	0.9800
Pt1—N1	2.015 (4)	C21—H21C	0.9800
Pt1—Cl1	2.3070 (10)	C22—H22A	0.9800
Pt1—Pt1^i^	3.2774 (3)	C22—H22B	0.9800
N1—C5	1.333 (6)	C22—H22C	0.9800
N1—C1	1.371 (7)	C23—H23A	0.9800
N2—C10	1.340 (5)	C23—H23B	0.9800
N2—C6	1.344 (5)	C23—H23C	0.9800
N3—C15	1.338 (6)	C24—C27	1.528 (7)
N3—C11	1.381 (5)	C24—C26	1.529 (6)
C1—C2	1.381 (7)	C24—C25	1.536 (7)
C1—C6	1.483 (7)	C25—H25A	0.9800
C2—C3	1.408 (8)	C25—H25B	0.9800
C2—H2A	0.9500	C25—H25C	0.9800
C3—C4	1.402 (8)	C26—H26A	0.9800
C3—C16	1.531 (7)	C26—H26B	0.9800
C4—C5	1.378 (7)	C26—H26C	0.9800
C4—H4A	0.9500	C27—H27A	0.9800
C5—H5A	0.9500	C27—H27B	0.9800
C6—C7	1.391 (6)	C27—H27C	0.9800
C7—C8	1.402 (6)	C1A—C2A	1.3900
C7—H7A	0.9500	C1A—C6A	1.3900
C8—C9	1.403 (6)	C1A—C7A	1.465 (6)
C8—C20	1.544 (6)	C2A—C3A	1.3900
C9—C10	1.393 (6)	C2A—H2AA	0.9500
C9—H9A	0.9500	C3A—C4A	1.3900
C10—C11	1.490 (5)	C3A—H3AA	0.9500
C11—C12	1.365 (6)	C4A—C5A	1.3900
C12—C13	1.404 (6)	C4A—H4AA	0.9500
C12—H12A	0.9500	C5A—C6A	1.3900
C13—C14	1.398 (6)	C5A—H5AA	0.9500
C13—C24	1.534 (6)	C6A—H6AA	0.9500
C14—C15	1.375 (7)	C7A—H7AA	0.9800
C14—H14A	0.9500	C7A—H7AB	0.9800
C15—H15A	0.9500	C7A—H7AC	0.9800
C16—C18	1.511 (11)	C1B—C2B	1.3900
C16—C19	1.530 (8)	C1B—C6B	1.3900
C16—C17	1.541 (8)	C1B—C7B	1.461 (6)
C17—H17A	0.9800	C2B—C3B	1.3900
C17—H17B	0.9800	C2B—H2BA	0.9500
C17—H17C	0.9800	C3B—C4B	1.3900
C18—H18A	0.9800	C3B—H3BA	0.9500
C18—H18B	0.9800	C4B—C5B	1.3900
C18—H18C	0.9800	C4B—H4BA	0.9500
C19—H19A	0.9800	C5B—C6B	1.3900
C19—H19B	0.9800	C5B—H5BA	0.9500
C19—H19C	0.9800	C6B—H6BA	0.9500
C20—C22	1.529 (7)	C7B—H7BA	0.9800
C20—C21	1.530 (7)	C7B—H7BB	0.9800
C20—C23	1.532 (7)	C7B—H7BC	0.9800
			
N2—Pt1—N3	80.87 (14)	C22—C20—C8	106.3 (4)
N2—Pt1—N1	81.25 (15)	C21—C20—C8	110.2 (4)
N3—Pt1—N1	162.06 (16)	C23—C20—C8	110.8 (4)
N2—Pt1—Cl1	178.70 (11)	C20—C21—H21A	109.5
N3—Pt1—Cl1	99.11 (10)	C20—C21—H21B	109.5
N1—Pt1—Cl1	98.72 (12)	H21A—C21—H21B	109.5
C5—N1—C1	119.1 (4)	C20—C21—H21C	109.5
C5—N1—Pt1	127.4 (3)	H21A—C21—H21C	109.5
C1—N1—Pt1	113.4 (3)	H21B—C21—H21C	109.5
C10—N2—C6	123.7 (4)	C20—C22—H22A	109.5
C10—N2—Pt1	118.5 (3)	C20—C22—H22B	109.5
C6—N2—Pt1	117.8 (3)	H22A—C22—H22B	109.5
C15—N3—C11	118.5 (4)	C20—C22—H22C	109.5
C15—N3—Pt1	127.4 (3)	H22A—C22—H22C	109.5
C11—N3—Pt1	114.0 (3)	H22B—C22—H22C	109.5
N1—C1—C2	121.2 (5)	C20—C23—H23A	109.5
N1—C1—C6	114.4 (4)	C20—C23—H23B	109.5
C2—C1—C6	124.4 (5)	H23A—C23—H23B	109.5
C1—C2—C3	120.2 (5)	C20—C23—H23C	109.5
C1—C2—H2A	119.9	H23A—C23—H23C	109.5
C3—C2—H2A	119.9	H23B—C23—H23C	109.5
C4—C3—C2	116.8 (5)	C27—C24—C26	108.7 (4)
C4—C3—C16	122.9 (5)	C27—C24—C13	107.4 (4)
C2—C3—C16	120.2 (5)	C26—C24—C13	112.0 (4)
C5—C4—C3	120.3 (5)	C27—C24—C25	109.0 (4)
C5—C4—H4A	119.9	C26—C24—C25	110.1 (4)
C3—C4—H4A	119.9	C13—C24—C25	109.6 (4)
N1—C5—C4	122.3 (5)	C24—C25—H25A	109.5
N1—C5—H5A	118.8	C24—C25—H25B	109.5
C4—C5—H5A	118.8	H25A—C25—H25B	109.5
N2—C6—C7	119.1 (4)	C24—C25—H25C	109.5
N2—C6—C1	113.1 (4)	H25A—C25—H25C	109.5
C7—C6—C1	127.8 (4)	H25B—C25—H25C	109.5
C6—C7—C8	119.3 (4)	C24—C26—H26A	109.5
C6—C7—H7A	120.3	C24—C26—H26B	109.5
C8—C7—H7A	120.3	H26A—C26—H26B	109.5
C7—C8—C9	119.3 (4)	C24—C26—H26C	109.5
C7—C8—C20	120.6 (4)	H26A—C26—H26C	109.5
C9—C8—C20	120.1 (4)	H26B—C26—H26C	109.5
C10—C9—C8	119.1 (4)	C24—C27—H27A	109.5
C10—C9—H9A	120.5	C24—C27—H27B	109.5
C8—C9—H9A	120.5	H27A—C27—H27B	109.5
N2—C10—C9	119.4 (4)	C24—C27—H27C	109.5
N2—C10—C11	112.9 (4)	H27A—C27—H27C	109.5
C9—C10—C11	127.7 (4)	H27B—C27—H27C	109.5
C12—C11—N3	121.2 (4)	C2A—C1A—C6A	120.0
C12—C11—C10	125.2 (4)	C2A—C1A—C7A	124.9 (5)
N3—C11—C10	113.6 (4)	C6A—C1A—C7A	115.1 (5)
C11—C12—C13	120.8 (4)	C3A—C2A—C1A	120.0
C11—C12—H12A	119.6	C3A—C2A—H2AA	120.0
C13—C12—H12A	119.6	C1A—C2A—H2AA	120.0
C14—C13—C12	116.6 (4)	C4A—C3A—C2A	120.0
C14—C13—C24	122.7 (4)	C4A—C3A—H3AA	120.0
C12—C13—C24	120.7 (4)	C2A—C3A—H3AA	120.0
C15—C14—C13	120.5 (4)	C3A—C4A—C5A	120.0
C15—C14—H14A	119.7	C3A—C4A—H4AA	120.0
C13—C14—H14A	119.7	C5A—C4A—H4AA	120.0
N3—C15—C14	122.2 (4)	C4A—C5A—C6A	120.0
N3—C15—H15A	118.9	C4A—C5A—H5AA	120.0
C14—C15—H15A	118.9	C6A—C5A—H5AA	120.0
C18—C16—C19	109.3 (6)	C5A—C6A—C1A	120.0
C18—C16—C3	109.6 (5)	C5A—C6A—H6AA	120.0
C19—C16—C3	111.1 (5)	C1A—C6A—H6AA	120.0
C18—C16—C17	110.2 (5)	C2B—C1B—C6B	120.0
C19—C16—C17	109.3 (6)	C2B—C1B—C7B	120.3 (5)
C3—C16—C17	107.4 (4)	C6B—C1B—C7B	119.7 (5)
C16—C17—H17A	109.5	C1B—C2B—C3B	120.0
C16—C17—H17B	109.5	C1B—C2B—H2BA	120.0
H17A—C17—H17B	109.5	C3B—C2B—H2BA	120.0
C16—C17—H17C	109.5	C4B—C3B—C2B	120.0
H17A—C17—H17C	109.5	C4B—C3B—H3BA	120.0
H17B—C17—H17C	109.5	C2B—C3B—H3BA	120.0
C16—C18—H18A	109.5	C3B—C4B—C5B	120.0
C16—C18—H18B	109.5	C3B—C4B—H4BA	120.0
H18A—C18—H18B	109.5	C5B—C4B—H4BA	120.0
C16—C18—H18C	109.5	C4B—C5B—C6B	120.0
H18A—C18—H18C	109.5	C4B—C5B—H5BA	120.0
H18B—C18—H18C	109.5	C6B—C5B—H5BA	120.0
C16—C19—H19A	109.5	C5B—C6B—C1B	120.0
C16—C19—H19B	109.5	C5B—C6B—H6BA	120.0
H19A—C19—H19B	109.5	C1B—C6B—H6BA	120.0
C16—C19—H19C	109.5	C1B—C7B—H7BA	109.5
H19A—C19—H19C	109.5	C1B—C7B—H7BB	109.5
H19B—C19—H19C	109.5	H7BA—C7B—H7BB	109.5
C22—C20—C21	109.1 (5)	C1B—C7B—H7BC	109.5
C22—C20—C23	111.5 (5)	H7BA—C7B—H7BC	109.5
C21—C20—C23	108.9 (4)	H7BB—C7B—H7BC	109.5

Symmetry codes: (i) −*x*+1, −*y*+1, −*z*+1.
